# Examining the Relationship of Clergy Distress, Spiritual Well-Being, Stress Management and Irritation to Life Satisfaction among Black Pastors in the USA

**DOI:** 10.1007/s10943-022-01715-1

**Published:** 2022-12-13

**Authors:** Robert C. Rogers

**Affiliations:** 1 Department of Counseling, Montclair State University, Montclair, NJ USA; 2 Church of God in Christ, PO Box 1532, Morristown, NJ 07962-1532 USA

**Keywords:** Occupational stress, Black pastor, Clergy distress, Black church

## Abstract

This study sought to determine the level of clergy distress and other psychological characteristics of Black pastors and their relationship to life satisfaction through a convenience sample of 2786 Black pastors in historically Black Protestant denominations and nondenominational Black churches. The response rate equaled 10.1% (283/2786) while the survey completion rate equaled 77% (218/283). These 218 Black pastors were serving as either senior pastors (86.3%) or co-pastors (13.7%). This study found clergy distress in Black pastors did not differ based on gender or age but differed by church size and denomination. Clergy distress (*r* =  − .187, *p* = .023) and irritation (*r* =  − .293, *p* = .003) possessed significant relationships with satisfaction with life as expected, but stress management (*r* = .039, *p* = .641), spiritual well-being in daily life (*r* = .140, *p* = .140), and spiritual well-being in ministry (*r* =  − .064, *p* = .475) did not, which was surprising. Notably strong relationships existed between stress management and spiritual well-being in daily life (*r* = .469, *p* = .003) and stress management and irritation (*r* =  − .359, *p* = .003). These two important relationships may offer some guideposts for Black pastors in developing strategies to combat the impact of both clergy distress and irritation. The study concludes with implications for Black pastors and suggestions for future research.

## Introduction

Pastors are spiritual and religious leaders who have been officially commissioned by religious organizations to perform religious/spiritual services. Pastors provide leadership for communities of faith as governed by their respective religious institutions and denominational structures and perform various roles within churches and communities depending upon their size, location, needs, and dynamics (Adams et al., 2017; Carroll, 2006). Carroll (2006) described the work of pastors as a "tough, demanding job, one that is not always very well understood or appreciated" and "more complex" than what pastors do during the Sunday worship service (p. 2).

Researchers have described pastors as one of several occupations for which stress exists as an integral part of the job (Adams et al., 2017; Darling et al., 2004; Hill et al., 2003). Adams et al. (2017) found the pastoral profession similar to social work, counseling, and teaching through their high levels of emotional involvement and interpersonal engagement. Further, they described a similar comparison with emergency personnel and police in that all three professions experienced schedule unpredictability, frequent crisis response, and stress-induced physiological arousal. Moreover, pastors reported “a similar level of EE (emotional exhaustion) as social workers, counselors, and emergency personnel and a lower level than teachers or police officers” (Adams et al., 2017, p. 166).

Carroll (2006) acknowledged that churches, regardless of denominational affiliation, can be both challenging and satisfying places for pastors to work. Interpersonal dynamics, conflicts, demands and expectations people may hold, heavy workloads, lack of social support, and emotional processes within a congregation and surrounding community all contribute to pastoral stress (Ford et al., 2014; Son, 2019). Yet, pastors have tended to focus more on taking care of others before taking care of themselves by denying, minimizing, or postponing their needs for self-care, which may increase their risk of being negatively impacted by stress and their proneness to burnout (Chandler, 2009; Doolittle, 2010). Pastoral stress negatively influences the emotional, mental, and physical wellness of pastors by contributing to psychological and physical strain (Darling et al., 2004; Wells, 2013). The stressors of pastoral ministry may affect pastors in different ways and, in the most extreme, may include burnout, leaving the ministry, or suicide (Bailey, 2019; Doolittle, 2010; Turton & Francis, 2007). The strategies pastors implement to cope with stress will affect their level/intensity of pastoral stress and influence their wellness and quality of life (Lazarus & Folkman, 1984; Webb & Chase, 2019).

Hill (1949) conceptualized Family Stress Theory with the A + B + C = X model, which served as a theoretical framework in clergy stress studies by Darling et al. (2004) as well as Lee and Iverson-Gilbert (2003). The initial stressor event (A), which challenges a person's ability to meet the event's demands, may have its source in the workplace, family, community, or the persons themselves. The individual's or family's coping resources (B) are intended to meet the demands of the stressor event, avoid a further crisis, and may include problem-solving skills, financial resources, seeking needed expertise, and stress management (Hill, 1949). The C-factor, which may be the most important variable in this model, represents the meaning and interpretation that the person gives the stressor event and their coping resources (Hill, 1949). The C-factor, more so than the stressor event itself, fuels the stress response. Therefore, the combination and interaction of the stressor event (A), coping resources (B), and meaning and interpretation (C) all contribute to an outcome, X, which may be negative or positive.

Researchers have investigated pastoral stress from a color-blind, etic perspective without regard to racial identity and ethnicity. Since the eighteenth century, Black pastors have provided visionary, organizational, and spiritual leadership for Black churches to meet the diverse needs of church and community members and contribute to Black people’s survival and well-being (Gates, 2021; Mamiya, 2005). While data from the Conference of National Black Churches (CNBC, n.d.) acknowledged that Black pastors have been a major subset of pastors nationwide, the stressors that Black pastors experience have been rarely studied with few exceptions. Mamiya (2005) and Carroll (2006) incorporated Black pastors from historically Black denominations in their research, while Smith (2013) focused her research on Black female pastors. Finally, Wimberley (2016) examined depression in a small sample of Black pastors. Pastoral stress may pose a threat to Black pastors’ well-being and warrants investigation.

There is much research across White denominations and denominational-specific populations concerning the stressors pastors experience. However, both past and more recent research has continued to focus on samples of primarily White, male pastors in White mainline and conservative Protestant denominations with only a small subset of pastors of color (Hough et al., 2019; Terry & Cunningham, 2020; Webb & Chase, 2019). Darling et al. (2004), as well as Hill et al. (2003), acknowledged the importance of future research including populations with various ethnic and racial backgrounds.

The purpose of this study was to fill the gaps in the literature by targeting a sample of Black pastors from historically Black denominations and nondenominational Black churches. More specifically, the researcher sought to answer two questions: What occupational stressors do Black pastors experience? What is the relationship between occupational stressors and the mental and emotional wellness among Black pastors? To ground the exploration of the research questions, the researcher hypothesized that (1) stress management and spiritual well-being will be significantly related to occupational stress and psychological strain, (2) Black women pastors will report higher occupational stress levels than Black male pastors, (3) Black pastors under 45 years will report higher occupational stress than Black pastors over 45 years, and (4) Black pastors of small churches (1–100 members) will report higher occupational stress than Black pastors of medium (101–350 members) and large churches (351+ members; Rogers, 2022).

## Method

### Sample

The study was reviewed and approved by the University’s Institutional Review Board. The sample was comprised of Black clergy, who were either senior pastors or co-pastors, working full-time or part-time in a predominantly Black church where the average weekly attendance was at least 75% Black. These Black pastors served a Black congregation that was either affiliated with a historically Black denomination or nondenominational. An a priori power analysis using G*Power with an alpha level of 0.05, a minimum power of 0.80, and a moderate effect size of 0.15 revealed a required sample size of at least 92 participants to find a statistically significant result. The survey response rate equaled 10.1% where 2786 Black pastors received the survey link and 283 participated in taking the survey. Of the 283 Black pastors who participated in the study, 218 successfully completed the survey in its entirety. Informed consent was obtained from all participants.

Table 1 depicts the demographic characteristics of the sample by gender, age, marital status, denomination, and employment status. Most pastors (87.1%, *n* = 190) worked between 1 and 50 h per week, with a mode of 1–20 h and a median of 20–30 h. Three quarters of participants (75.2%, *n* = 164) served in small churches, 19.3% (*n* = 42) in medium churches, and 5.5% (*n* = 12) in large churches (Rogers, 2022).

**Table 1:** Participant demographic characteristics

	*N*	%
Gender		
Male	135	61.9
Female	83	38.1
Age		
18–34 years	7	3.2
35–44 years	18	8.3
45–64 years	116	53.2
65 years and older	75	34.4
Marital status		
Married	154	70.6
Single	33	15.1
Divorced	23	10.6
Widowed	3	1.4
Separated	3	1.4
Prefer not to answer	2	0.9
Denomination		
Methodist	58	26.6
Church of god in Christ	57	26.1
Baptist	35	16.1
Pentecostal	26	11.9
Independent/non-denominational	25	11.5
Holiness/apostolic	10	4.6
Other	7	3.2
Employment status		
Full-time senior pastor	146	67.0
Full-time co-pastor	11	5.0
Part-time senior pastor	42	19.3
Part-time co-pastor	19	8.7

### Data Collection

The sample of Black pastors was recruited through a few sources: LinkedIn, historically Black denominations, How Shall They Hear Preaching Conference, the Church Of God In Christ Shepherds Conference, and word of mouth through Black pastors and church members. Black pastors on LinkedIn were invited over an 8-week period to participate in the study through a series of posted flyers and a video, all approved by the University IRB, containing an online link to the study. Black denominations received emails and phone calls to inform them of the study and requested they share the study flyer with their pastors. The denominations contacted were: National Baptist Convention USA, National Baptist Convention of America, National Missionary Baptist Convention, Progressive National Baptist Convention, Church Of God In Christ, Full Gospel Baptist Church Fellowship, African Methodist Episcopal Church, African American Episcopal Zion Church, Christian Methodist Episcopal Church, United Church of Deliverance, Apostolic Assemblies of Christ, and National Association of the Church of God.

The How Shall They Hear Conference, an annual preaching conference based in New Jersey which attracts Black pastors from the mid-Atlantic region, emailed the study’s flyer to Black pastors on their mailing list. The Church Of God In Christ Shepherds Conference was an online conference at which I introduced the study and invited pastors to participate. Therefore, a combination of convenience sampling and snowball sampling was used to seek participants. First, convenience sampling led to the survey information being emailed to pastors within various denominations and through pastors' networks. In addition, church members shared the survey flyer with their pastors, pastor friends, and relatives who were pastors. Second, some pastors who took the survey themselves shared the survey flyer with pastor friends and colleagues in a snowball-like fashion. However, there is no evidence on how the actual participants received the survey information and which distribution method was more effective in reaching potential participants (Rogers, 2022).

### Instruments

#### Clergy Occupational Distress Index (CODI)

The Clergy Occupational Distress Index (CODI) is a 5-question instrument which measures how often pastors experience occupational stress in the form of excessive demands, personal criticism, loneliness, isolation, and challenges from (1) Never, (2) Once in a while, (3) Fairly often, and (4) Very often (Frenk et al., 2013). Scores may range from 5 to 20 with higher scores indicating more occupational distress. Frenk et al. (2013) assessed construct validity using Carroll’s (2006) diverse clergy sample, which revealed Cronbach's alphas of 0.77 (Pulpit and Pew sample) and 0.82 (Clergy Health Study sample), both of which indicated good reliability.

#### Stress Management Subscale: Health-Promoting Lifestyle Profile II

The Stress Management Subscale is an 8-item instrument measuring the frequency of specific, proactive behaviors directed towards managing and reducing stress (Walker & Hill-Polerecky, 1996). Using a scale of (1) Never, (2) Sometimes, (3) Often, and (4) Routinely, the score on this subscale can range from 8 to 32 with higher scores meaning the more frequent practice of stress management, health-promoting behaviors (Walker & Hill-Polerecky, 1996). Further, Walker and Hill-Polerecky (1996) determined the psychometric properties of the HPLP-II from a study of 712 adults and using factor analysis to confirm the construct validity of the six subscales with the Stress Management Subscale demonstrating a Cronbach’s alpha of 0.79.

#### Clergy Spiritual Well-Being Scale

The Clergy Spiritual Well-being Scale assesses closeness to God specifically in clergy with the use of two sub-scales: Presence and Power of God in Daily Life and Presence and Power of God in Ministry (Proeschold-Bell et al., 2014). These sub-scales assume that closeness to God can vary over time, differ in one's daily life experiences versus church work, and "the frequency of experiencing God's power and presence would provide an indication of how close one feels to God" (Proeschold-Bell et al., 2014, p. 882). Each sub-scale contains six items and participants choose between five responses: (1) Never, (2) Sometimes, (3) Often, (4) Frequently, or (5) Always, with scores on each sub-scale ranging from 6 to 30 whereby higher scores indicate greater closeness to God and spiritual well-being. Proeschold-Bell et al. (2014) found that the two sub-scales were highly correlated at two different time points at 0.83 and 0.78 in a sample of 1,513 United Methodist Church clergy. Additively, the researchers found concurrent validity between the two sub-scales and measures for depression, anxiety, stress, emotional exhaustion, and depersonalization and a second group of measures for quality of life, ministry satisfaction, and personal accomplishment (Proeschold-Bell et al., 2014).

#### The Irritation Scale

The Irritation Scale is an 8-item survey which measures psychological strain in workplace settings in the form of irritation when individuals perceive obstacles to and uncertainty about important work goals (Mohr et al., 2006). Irritation includes both cognitive and emotional irritation where cognitive irritation manifests itself in rumination through which individuals repeatedly think about their work, especially problems and lack of goal attainment, outside of the work context (Mohr et al., 2006). On the other hand, emotional irritation presents more as an emotionally reactive response, which Mohr et al. (2006) described as irritability including elements of anger, annoyance, impatience, and frustration at the lack of goal attainment.

Both cognitive and emotional irritation may lead to mental strain, negative mood, and negative emotions, such as anxiety, depression, and lower self-esteem (Mohr et al., 2006). Participants rate their agreement with each of eight statements on a 7-point scale from (1) Strongly disagree to (7) Strongly agree. Scores range from 8 to 56 with higher scores indicating more irritation. Mohr et al. (2006) reported that the cognitive and emotional irritation subscales had internal consistency ranging from 0.85 to 0.97 and the total scale demonstrated positive correlation with such constructs as "psychosomatic complaints, depression, social stressors, emotional exhaustion, and depersonalization" (p. 199).

#### The Riverside Life Satisfaction Scale

The Riverside Life Satisfaction Scale (RLSS) is a 6-item measure assessing one's satisfaction with life, which included not only positive statements of contentment with one’s life but also statements of envy, regret, and wanting change on a 7-point scale ranging from (1) Strongly disagree to (7) Strongly agree (Margolis et al., 2019). Scores range from 6 to 42 with higher scores reflecting higher satisfaction with life. The RLSS correlated with the Satisfaction with Life Scale (Diener et al., 1985), the leading life satisfaction measurement tool since 1985, between 0.85 and 0.90 (Margolis et al., 2019).

### Statistical Analyses

The researcher analyzed and summarized the data using SPSS and used multiple regression to analyze the relationships among variables. Applying Hill’s (1949) Family Stress Theory A + B + C = X model to this study, we have: A (clergy distress) + B1 (stress management) + B2 (spiritual well-being in daily life) + B3 (spiritual well-being in ministry) + C (irritation as psychological strain) = X (satisfaction with life). The predictor variables were clergy distress, stress management, spiritual well-being in daily life, spiritual well-being in ministry, and irritation. The outcome variable was satisfaction with life (Rogers, 2022).

Hierarchical regression and path analysis explored the relationships among these variables in accordance with the Family Stress Theory A + B + C = X model. Preliminary analyses were performed to satisfy the assumptions for multiple regression. The IBM SPSS AMOS software was used to conduct a path analysis to further explore the relationships of the variables in this study based on the Family Stress Theory A + B + C = X model. The goal of this analysis was to assess any direct and indirect effects of variables, specifically if coping strategies mediated the effects of clergy distress and irritation (Rogers, 2022).

A one-way between-groups analysis of covariance (ANCOVA) was conducted to assess any differences between groups of Black pastors in clergy distress based on gender, age, and church size. The dependent (outcome) variable was clergy distress and the covariates were stress management, spiritual well-being in daily life, spiritual well-being in ministry, irritation, and satisfaction with life. The analysis procedure partitioned the variance of the dependent variable and accounted for the variance in the independent variables. The assumptions for ANCOVA were reviewed and met (Rogers, 2022).

## Results

### Data Analysis

The clergy distress mean score of 11.25 (std. deviation = 3.34) indicated that Black pastors experienced stress in the form of excessive demands, criticism, loneliness, isolation, and challenges on average at least "once in a while" (score = 2) but somewhat less than "fairly often" (score = 3). A one-sample *t* test evaluated whether this sample mean for Black pastors was significantly different than the test value population mean of 10.98 from the Pulpit & Pew Study sample of a nationally representative group of clergy (Frenk et al., 2013). The one-sample *t* test revealed that, while Black pastors had a slightly higher clergy distress score (mean difference = 0.26771), there was no significant difference between Black pastors and the Pulpit & Pew sample,* t* (217) = 1.183, *p* = 0.238 (Rogers, 2022).

The stress management mean score (19.40, std. deviation = 4.10) revealed that Black pastors “sometimes” (score = 2) practice specific behaviors to manage and reduce their stress more so than “often” (score = 3) or “routinely” (score = 4). The spiritual well-being in daily life score (22.13, std. deviation = 4.40) and spiritual well-being in ministry score (22.10, std. deviation = 4.92) both indicated that Black pastors experience closeness to God at least “often” (score = 3) but not quite “frequently” (score = 4) on average in their daily lives and during ministry activities. The irritation score (28.31, std. deviation = 11.19) indicated that Black pastors at least slightly disagreed (score = 3) to partially feeling neutral (“neither agree or disagree”; score = 4) that clergy distress was negatively affecting them both mentally and emotionally. The satisfaction with life score (28.58, std. deviation = 7.84) reflected that Black pastors tended to slightly agree (score = 5 versus moderately agree with a score = 6) with being satisfied with their lives (Rogers, 2022).

### Relationship Among Variables

In the hierarchical regression, the demographic variables of gender, age, marital status, living with family members, years as pastor, employment status, and denomination were entered in Step 1, explaining 3% of the variance in satisfaction with life. Clergy distress was entered at Step 2, explaining 13.3% of the variance in satisfaction with life. Within Step 3 stress management, spiritual well-being in daily life, and spiritual well-being in ministry were entered and explained an additional 4.7% of the variance. Within Step 4 irritation was entered, explaining an additional 4.6% of the variance. After Step 4, the total variance of satisfaction with life explained by the model as a whole was 25.6%. Reflecting significant *R* change, Step 1 was at *p* = 0.476, Step 2 at *p* < 0.001, Step 3 at *p* = 0.008, and Step 4 at *p* < 0.001.

The ANOVA indicated that the model as a whole was significant, F (12, 205) = 5.882, *p* < 0.001. In the final model only two variables were statistically significant and made a unique contribution to predict satisfaction with life with irritation recording a higher semipartial correlation (*sr* =  − 0.215, *p* < 0.001) than clergy distress score (*sr* =  − 0.147, *p* = 0.016). The coefficients table provided the unstandardized regression coefficients *B* and a Constant value (36.7, *p* < 0.001). The unexpected result in this table was the negative relationship between spiritual well-being in ministry (*B* =  − 0.165) and satisfaction with life when it was expected to be positive. See Table 2 for the hierarchical regression summary.

**Table 2:** Hierarchical Regression Summary, Satisfaction with Life as Outcome Variable

Step and predictor variables	*B*	*SE B*	Beta	Semi partial correlation	Change in *R*^2^	*R*^2^
Step 1					0.030	0.030
Constant	25.25	3.71				
Gender	− 0.75	1.21	− 0.05	− 0.04		
Age range^a^	1.91	0.87	0.16	0.15		
Marital status	0.01	0.69	0.001	0.001		
Live with family	− 0.46	1.11	− 0.03	− 0.03		
Years as pastor	− 0.82	0.76	− 0.09	− 0.07		
Employment status	− 0.45	0.54	− 0.06	− 0.06		
Denomination	0.17	0.26	0.05	0.04		
Step 2					0.133	0.163
Constant	39.57	4.26				
Gender	− 0.37	1.13	− 0.02	− 0.02		
Age range	0.59	0.84	0.05	0.04		
Marital status	0.09	0.64	0.01	0.01		
Live with family	− 0.84	1.04	− 0.05	− 0.05		
Years as pastor	− 0.48	0.71	− 0.05	− 0.04		
Employment status	− 0.86	0.51	− 0.12	− 0.11		
Denomination	0.09	0.24	0.03	0.02		
Clergy distress^b^	− 0.90	0.16	− 0.38	− 0.36		
Step 3					0.047	0.210
Constant	29.27	5.30				
Gender	− 0.67	1.11	− 0.04	− 0.04		
Age range	0.36	0.83	0.03	0.03		
Marital status	0.26	0.63	0.03	0.02		
Live with family	− 0.51	1.02	− 0.03	− 0.03		
Years as pastor	− 0.43	0.69	− 0.04	− 0.04		
Employment status	− 0.92	0.51	− 0.12	− 0.11		
Denomination	0.15	0.24	0.04	0.04		
Clergy distress^c^	− 0.76	0.16	− 0.32	− 0.29		
Stress management	0.27	0.14	0.14	0.11		
Spiritual WB daily	0.29	0.17	0.16	0.10		
Spiritual WB						
Ministry	− 0.11	0.14	− 0.07	− 0.05		
Step 4					0.046	0.256
Constant	36.70	5.56				
Gender	− 0.84	1.08	− 0.05	− 0.05		
Age range	0.38	0.81	0.03	0.03		
Marital status	− 0.07	0.62	− 0.01	− 0.01		
Live with family	0.22	1.01	0.01	0.01		
Years as pastor	− 0.47	0.67	− 0.05	− 0.04		
Employment status	− 0.91	0.49	− 0.12	− 0.11		
Denomination	0.22	0.23	0.06	0.06		
Clergy distress^d^	− 0.44	0.18	− 0.19	− 0.15		
Stress management	0.07	0.15	0.04	0.03		
Spiritual WB daily	0.29	0.17	0.16	0.10		
Spiritual WB						
Ministry	− 0.16	0.14	− 0.10	− 0.07		
Irritation^d^	− 0.21	0.60	− 0.30	− 0.21		

Dependent Variable: Satisfaction with Life

^a^Age Range, *p* = .029 (Model 1)

^b^Clergy Distress, *p* < .001 (Model 2)

^c^Clergy Distress, *p* < .001 (Model 3)

^d^Clergy Distress, *p* = .016; Irritation, *p* < .001 (Model 4)

### Path Analysis

**Fig. 1:**
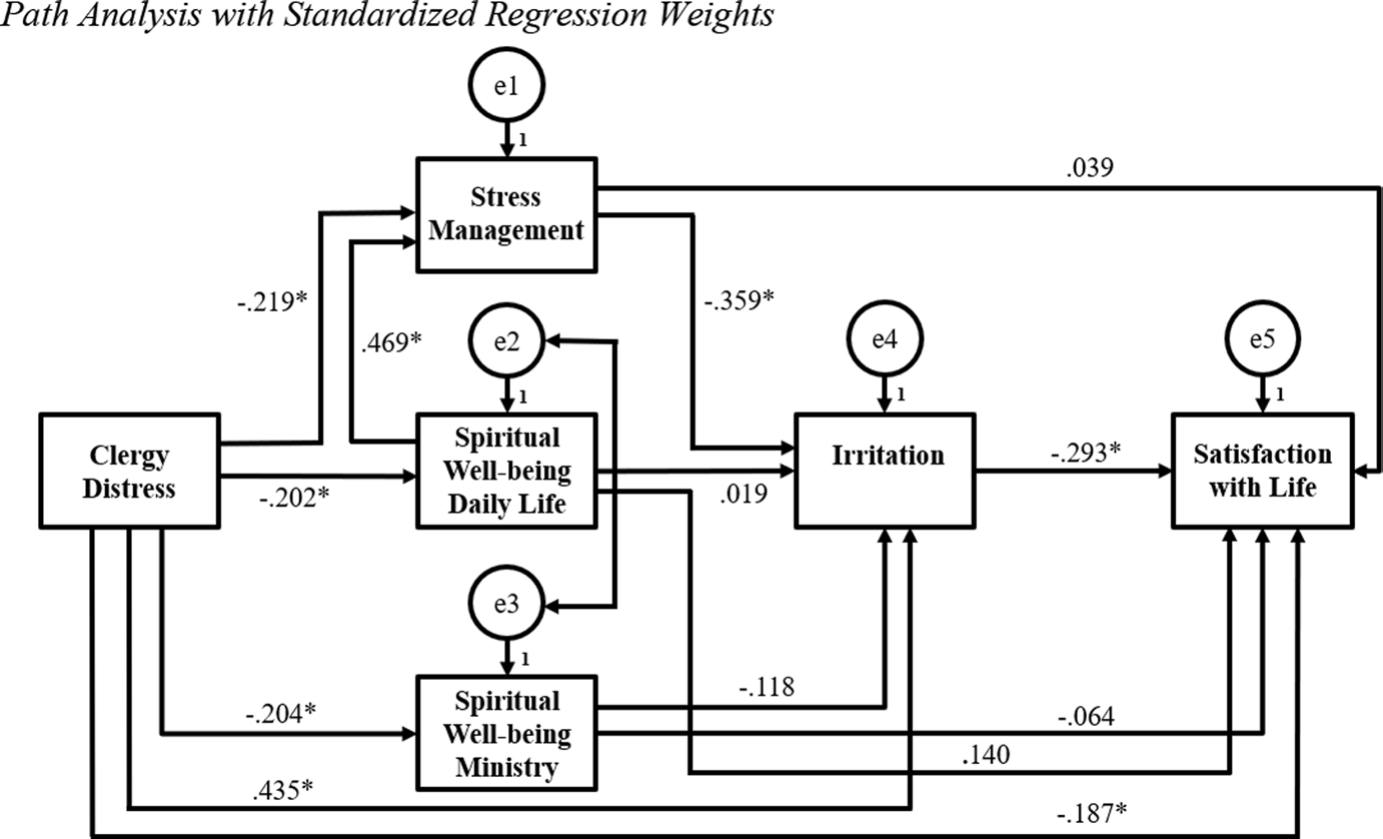
Path analysis with standardized regression weights. *Note.* The lines between the variables represent the relationship between variables. * indicates that the relationship is significant at *p* < .05. e1, e2, e3, e4, and e5 are residual terms indicating the error in measuring the relationship between clergy distress as the exogenous (predictor) variable and the five endogenous (outcome) variables. The strongest relationships existed between: (1) stress management and spiritual well-being in daily life (*r* = .469, *p* = .003), (2) clergy distress and irritation (*r* = .435, *p* = .002), (3) stress management and irritation (*r* =  − .359, *p* = .003), and (4) irritation and satisfaction with life (*r* =  − .293, *p* = .003)

Figure 1 shows the path model with the standardized regression weights with an asterisk indicating a significant relationship. The path model depicts the role and impact of the coping strategy variables (i.e., stress management, spiritual well-being in daily life, and spiritual well-being in ministry) in relation to clergy distress, irritation, and satisfaction with life. Clergy distress had a significant inverse relationship with spiritual well-being in daily life (*r* =  − 0.202, *p* = 0.006), spiritual well-being in ministry (*r* =  − 0.204, *p* = 0.003), and stress management (*r* =  − 0.219, *p* = 0.002). Of the three coping variables, only stress management had a significant relationship with irritation (*r* =  − 0.359, *p* = 0.003). In addition, stress management and spiritual well-being in daily life possessed a significant, strong relationship (*r* = 0.469, *p* = 0.003).

None of the coping variables had a significant relationship with satisfaction with life: stress management (*r* = 0.039, *p* = 0.641), spiritual well-being in daily life (*r* = 0.140, *p* = 0.140), and spiritual well-being in ministry (*r* =  − 0.064, *p* = 0.475). Clergy distress had a significant inverse relationship with satisfaction with life (*r* =  − 0.187, *p* = 0.023) whereby higher levels of occupational stress resulted in lower satisfaction with life. Further, clergy distress possessed a significant, strong relationship with irritation (*r* = 0.435, *p* = 0.002). Moreover, irritation had a significant inverse relationship with satisfaction with life (*r* =  − 0.293, *p* = 0.003), meaning that higher irritation resulted in lower satisfaction with life. In sum, Hypothesis 1 was partially supported by all three coping variables having a significant inverse relationship with clergy distress. However, only stress management had a significant inverse relationship with irritation, which provided a mediated, indirect path from clergy distress to irritation (Rogers, 2022).

### Differences Between Groups

After adjusting for the covariates, no significant difference was found between Black male and female pastors on the clergy distress score, *F* (1, 211) = 0.027, *p* = 0.870, partial eta squared = 0.00. In addition, there was no significant difference between pastors in age, *F* (3, 209) = 2.169, *p* = 0.093, partial eta squared = 0.03. There was a significant difference found in clergy distress based on church size, *F* (2, 210) = 3.874, *p* = 0.022, partial eta squared = 0.036. After adjusting for the influence of the covariates, the following were the means for pastors based on church size: 10.96, std. error = 0.212 (small churches), 11.98, std. error = 0.421 (medium churches), and 12.61, std. error = 0.799 (large churches). Further, pastors in large churches (*p* = 0.048) and medium churches (*p* = 0.032) both had a significantly higher clergy distress score compared to pastors in small churches. Interestingly, there was no significant difference between pastors in large and medium-sized churches (*p* = 0.491). Therefore, Hypothesis 2 and Hypothesis 3 were not supported by the data. However, the data did reveal a difference in clergy distress based on church size (Hypothesis 4; Rogers, 2022).

When comparing denominations, there was a significant difference found in the clergy distress score, *F* (6, 206) = 2.732, *p* = 0.014, partial eta squared = 0.074. After adjusting the means for the effects of the covariates, the clergy distress scores from lowest to the highest were Methodist (10.438, std. error = 0.353), Church Of God In Christ (10.835, std. error = 0.357), Pentecostal (11.718, std. error = 0.530), Independent/Non-denominational/Other (11.748, std. error = 0.477), and Baptist (12.399, std. error = 0.460). The Baptists had a significantly higher clergy distress score than Church Of God In Christ (*p* = 0.008) and Methodists (*p* < 0.001). Additionally, the Methodists had a significantly lower score than the Pentecostals (*p* = 0.046) and Independent/Non-denominational/Other (*p* = 0.029; Rogers, 2022).

## Discussion

Both the hierarchical regression and path analysis confirmed the negative, significant relationships of clergy distress and irritation with satisfaction with life, which was expected. The path analysis further identified the strong positive relationship between clergy distress and irritation, also expected, and which manifested as the second strongest relationship among variables. Clergy distress directly feeds into and sustains irritation so that Black pastors may experience negative thoughts and feelings after leaving the workplace but with the workplace stressors continuing to occupy their attention. Similar to findings by Hoge (2009), such irritation may distract needed personal attention and presence from the pastor's family or her/his personal life. These relationships may provide Black pastors with an alert to be watchful on the potential influence clergy distress and irritation may have on their emotional and occupational wellness as well as satisfaction with life.

While the hierarchical regression revealed that the demographic variables, clergy distress, spiritual well-being, stress management, and irritation accounted for only 25.6% of the variance in satisfaction with life, it will be helpful to identify other factors which contribute to the additional unexplained variance. First, some other possible contributing factors might include (a) pastors' sense of calling from God, (b) support of the congregation in such forms as prayer, affirmations, respect, and volunteering, (c) support of family members and participation in pastoral/church work, (d) pastors' leadership style, and (e) work-life balance. Second, it may be important to explore what satisfaction with life means experientially for Black pastors. For example, pastors who work secular jobs in addition to pastoring a small church may hold a different existential meaning of satisfaction with life than those who pastor full-time in medium and large churches. Third, in addition to their work pastoral stressors, satisfaction with life may be influenced by personal stressors in relation to health and finances, living with and caring for family members, and societal stress like social injustice and racial microaggressions.

The path analysis revealed that all three coping strategies presented as important in combating clergy distress. Yet, none of the coping strategies had a significant, positive relationship with satisfaction with life. This was surprising and somewhat conflicting with previous literature, as it had been found that spiritual well-being significantly enhanced quality of life (Darling et al., 2004; Proeschold-Bell et al., 2015). However, there were differences in the relationships between the coping variables and irritation. Specifically, spiritual well-being in daily life and spiritual well-being in ministry did not have a significant inverse relationship with irritation. This was also an unexpected result and contrary to Darling et al. (2004) findings that spiritual well-being was a stronger protective factor than family coping resources against psychological and physiological stress. The results were further contrary to Proeschold-Bell et al. (2015) finding that spiritual well-being in daily life was a significant protective factor against depression, anxiety, emotional exhaustion, and depersonalization, while spiritual well-being in ministry similarly protected against depersonalization.

Most significantly, the results indicated that the strongest relationship between variables existed between stress management and spiritual well-being in daily life. Further, the third strongest relationship among variables existed between stress management and irritation, a significant inverse relationship. Yet, the spiritual well-being coping strategies did not possess a significant inverse relationship with irritation, which was surprising. In addition, stress management had a slightly stronger inverse relationship with clergy distress than spiritual well-being in daily life and spiritual well-being in ministry. While Black pastors may be more prone to focus on and practice their spiritual well-being, they may consider adding and/or increasing stress management in their efforts to combat both clergy distress and irritation.

The data indicated similarities in the pastoral stress that Black male and female pastors experience. The pastoral role has traditionally been held in high esteem and respect by Black church and community members, with role of pastor carrying certain responsibilities and expectations (Gates, 2021; Mamiya, 2005). The data affirmed that both female and male pastors experience clergy distress and irritation to a similar degree. These results support previous research that stress, particularly clergy distress, is endemic to the pastoral role, regardless of race or gender (Berry et al., 2012; Carroll, 2006; Frenk et al., 2013).

The overwhelming majority of Black pastors occupied the upper age ranges (i.e., 45–64 years and 65+ years). The percentage of Black pastors in this study being 65 years and older lends support to Carroll’s (2006) finding of Black denominations having the highest percentage of pastors over the age of 61 years compared to conservative and mainline Protestant denominations. Some Black pastors may continue to work as long as possible for financial reasons, such as lack of retirement funds (Carroll, 2006). Yet, other Black pastors may continue to work from a sense of a higher calling that there remains a purpose and work to accomplish.

The data confirmed the predominance of small churches where Black church members attend more than medium and large churches (Carroll, 2006; Pew Research Center, 2021). The original hypothesis that Black pastors in small churches would experience greater clergy distress due to wearing many hats, performing different roles, and having less privacy was not supported by the data. Yet, what proved interesting about these Black pastors in small churches was the range of hours they worked. While most pastors worked 1–20 h, 20–30 h, and 30–40 h per week, a small group of 11 pastors worked more than 50 h per week. Black pastors, working part-time in small churches which are not able to provide a full salary and benefits, experience financial stress requiring them to work additionally a secular job. While there may be similarities in the work of Black pastors in any size church, there may be unique experiential differences for those working in small churches, which may influence their pastoral stress and satisfaction with life.

The study's results showed an unexpected difference in clergy distress score based on denomination whereby Baptist pastors had the highest score while Methodist and COGIC pastors had the lowest scores. One factor may be that Baptist churches possess autonomy and independence where the power of governing lies within the congregation. Black Baptist pastors have to contend with the power of deacons and lay leaders, which may be stressful. Another factor is church size where the results indicated that pastors in medium and large churches had higher clergy distress than pastors in small churches. Baptist pastors served in more medium and large churches than other denominations in this study whereas at least 80% of Methodist and COGIC churches were small churches. This difference by denomination in clergy distress score is surprising and hard to fully explain without more investigation.

While this study confirmed that Black pastors experience clergy distress and irritation, it did not fully answer the research question on the relationship between clergy distress and the emotional and mental wellness of Black pastors. Resulting from their clergy distress, Black pastors sometimes experienced psychological strain in the forms of cognitive irritation and emotional irritation, especially during their leisure time. Hoge (2009) acknowledged that irritation may increase negative mood, decrease psychological functioning, may have a long-term connection to depression, and found a "strong direct effect from emotional irritation to psychosomatic complaints," such as headaches, dizziness, and digestive problems (p. 47). The present study was not able to assess such direct emotional, mental, or physical effects.

## Implications

The Family Stress Theory A + B + C = X model (Hill, 1949) described that, after stressor events (A) begin, individuals activate their coping strategies (B). Yet, from the study’s results, Black pastors’ effectiveness in successfully coping with their stressors remains questionable, especially with them reacting to the stressors they experience. The study’s results may suggest that a more proactive approach by Black pastors to successfully combat clergy distress and irritation as well as enhance satisfaction with life may be helpful. The path analysis indicated the strong relationship between stress management and spiritual well-being in daily life. Black pastors may consider using the combination of these coping strategies by working them collaboratively to affect both clergy distress and irritation. The incorporation of these two coping strategies together can become part of a self-care and wellness plan for Black pastors.

Black pastors must realize the importance of stress management to their wellness and make a daily commitment to implement stress management strategies. Yet, the need for stress management may be surprising to some Black pastors, especially those who hold beliefs of (1) enduring and persevering through stressor events without acknowledging or addressing them and (2) taking their problems, burdens, and issues only to God in prayer who they hope will solve them. As Black pastors commit daily time to spiritual well-being in the form of prayer, they must realize the important value of the combination of spiritual well-being and stress management, such as combining prayer and meditation with walking. Moreover, pastors cannot assume they are close to God because they work in a church. Rather, their relationship and closeness to God requires intentional awareness and consistent effort by increasing their frequency of experiencing the presence and power of God from “often” to at least “frequently.”

This study’s results seem to suggest the importance of Black pastors paying more attention to levels of clergy distress and irritation and their corresponding influence on the satisfaction with life. Even though this study did not find specific, measurable effects on Black pastors’ mental and emotional wellness, their experience of clergy distress and irritation may serve as a precautionary sign for more attention and greater awareness to prevent clergy distress and irritation from causing other psychological and physiological problems (Hoge, 2009). With increased awareness of the potential negative effects of clergy distress and irritation, Black pastors can seek the assistance of licensed professional counselors to help them accomplish their wellness, mental health, and career goals. For example, counseling may help pastors to examine their perceptions of stressor events to address any cognitive distortions, process their feelings, and explore different stress management strategies.

Black Church denominations must pay attention to the stressors their pastors experience, which may affect pastors’ ability to serve, meet church members’ needs, and lead their congregations effectively. Such attention can provide useful information to Black Church leaders regarding the stressors, challenges, and issues of being a Black pastor in today’s world. This knowledge can further equip Black Church leaders to promote the health and wellness of their pastors, which in turn may lead to healthier congregations.

## Limitations

There are several limitations to this study that should be mentioned. The study was conducted strictly through online data collection and during the COVID-19 pandemic, which limited contact with potential participants. While a random sample was desirable, the logistical and financial resources were not available to conduct this study to meet those requirements. Consequently, the sample size was small and the sample composition was not truly representative of the population of Black pastors nationwide. This study depended primarily on convenience sampling and, to a much lesser degree, on snowball sampling to achieve its sample size. While these methods proved useful in this study, they did not present as reliable methods of achieving a representative sample and adequate sample size. Thus, a larger and more representative sample would have been helpful.

Another limitation exists in the data collection methodology, specifically in the survey instruments used to collect the data. A shorter survey with fewer questions may have contributed to higher completion rates and sample size. While the CODI measured clergy distress, there may be other stressors that Black pastors experience, which a second instrument could have measured. Perhaps to better assess the effect of stress on pastors' mental and emotional wellness, an assessment tool specifically measuring mental health outcomes may have been helpful.

## Suggestions for Future Research

There are a few areas where future research can explore. First, identifying what other factors may contribute to satisfaction with life for Black pastors is important and should be further explored in qualitative and quantitative studies. Second, the question of why spiritual well-being did not have a significant relationship with satisfaction with life needs exploration. This lack of significant relationship found in this study is not an indication of the absence of spiritual well-being in Black pastors, rather it warrants a closer examination of these relationships. Third, with Black pastors in small churches occupying a majority within the population of Black pastors, a closer look at their experiences would be insightful. More specifically, future studies should look at clergy distress and other related stressors, and their relationships to satisfaction with life as well as quality of life. Fourth, an investigation into the differences in clergy distress based on denominations and the contributing factors would be advantageous. Finally, since prolonged exposure to stress without effective coping strategies may contribute to burnout, assessing the prevalence of burnout symptoms and how these symptoms are handled among Black pastors would be a worthwhile topic for future research.

## Conclusion

This study uncovered many insights into Black pastors and their experiences. Clergy distress and irritation had a significant inverse relationship with satisfaction with life. The coping strategies of stress management, spiritual well-being in daily life, and spiritual well-being in ministry all possessed significant inverse relationships with clergy distress, with stress management having the strongest relationship. None of the coping strategies contributed significantly to satisfaction with life and only stress management possessed a significant, inverse relationship with irritation. The strong, significant, positive relationship between spiritual well-being in daily life and stress management may be useful to Black pastors in their efforts to combat both clergy distress and irritation.

Based on the findings of this study, clergy distress seems to be endemic to the pastoral role regardless of race, gender, age, church size, years as pastor, and denomination. The results of this study contribute to the knowledge base of pastoral stress by providing an emic perspective of Black pastors in historically Black Protestant denominations and nondenominational Black congregations. This study fills a seemingly large gap in the literature by identifying the relationship between Black pastors’ clergy distress, irritation, and coping strategies and their satisfaction with life.
